# Integrated Multiple “-omics” Data Reveal Subtypes of Hepatocellular Carcinoma

**DOI:** 10.1371/journal.pone.0165457

**Published:** 2016-11-02

**Authors:** Gang Liu, Chuanpeng Dong, Lei Liu

**Affiliations:** Institutes of Biomedical Sciences, Fudan University, 200032, Shanghai, China; Taipei Medical University College of Medicine, TAIWAN

## Abstract

Hepatocellular carcinoma is one of the most heterogeneous cancers, as reflected by its multiple grades and difficulty to subtype. In this study, we integrated copy number variation, DNA methylation, mRNA, and miRNA data with the developed “cluster of cluster” method and classified 256 HCC samples from TCGA (The Cancer Genome Atlas) into five major subgroups (S1-S5). We observed that this classification was associated with specific mutations and protein expression, and we detected that each subgroup had distinct molecular signatures. The subclasses were associated not only with survival but also with clinical observations. S1 was characterized by bulk amplification on 8q24, TP53 mutation, low lipid metabolism, highly expressed onco-proteins, attenuated tumor suppressor proteins and a worse survival rate. S2 and S3 were characterized by telomere hypomethylation and a low expression of TERT and DNMT1/3B. Compared to S2, S3 was associated with less copy number variation and some good prognosis biomarkers, including CRP and CYP2E1. In contrast, the mutation rate of CTNNB1 was higher in S3. S4 was associated with bulk amplification and various molecular characteristics at different biological levels. In summary, we classified the HCC samples into five subgroups using multiple “-omics” data. Each subgroup had a distinct survival rate and molecular signature, which may provide information about the pathogenesis of subtypes in HCC.

## Introduction

Hepatocellular carcinoma (HCC) is the fifth leading cancer worldwide. It is also the third most common cause of death in all cancers [1]. HCC has multiple causal factors, including alcohol consumption, hepatitis B/C virus (HBV/HCV) infection and cirrhosis [2]; therefore, it is more heterogeneous than other cancer types and can have varying prognosis. Thus, it is important to classify patients into subgroups to enable precise therapy.

Over the past decade, efforts have been devoted to the molecular classification of HCC. These studies were mainly based on an analysis of genomic alterations, including somatic mutations and copy number variations [3]. Transcriptomic alterations consisting of gene expression changes and microRNA re-patterning [4, 5] were also identified. WNT [6], mTORC [7], and other important pathways in carcinogenesis have been detected and used for classification, and subclasses based on CTNNB and AXIN mutations have been identified. However, the clinical application of the HCC markers identified in previous studies is limited by the heterogeneous origins of carcinogenesis within the sample sets used in each respective study [8].

The alterations of cancer cells can occur on various levels, including somatic mutation, copy number variation (CNV) [9], methylation [10], transcription and miRNA [11, 12]. Studies have indicated that the integration of these factors improves HCC classification [13]. TCGA (http://cancergenome.nih.gov/) provides thousands of samples of various cancers, including data on somatic mutation, DNA methylation, miRNA-seq, copy number variation (CNV), and clinical observations. A lack of methods to integrate these data regarding different biological characteristics has posed a huge problem. In recent years, the method based on the “cluster of cluster” has proven to be a powerful way to detect heterogeneity during carcinogenesis [14, 15], and it is an effective method for cancer classification. It is independent of the feature number of each platform, and the contribution of each platform is determined by the cluster number of each biological level. However, the integration of multiple “-omics” data for HCC samples has not been conducted previously, and limitations exist in this method.

In this vein, we screened the landscape of DNA methylation, copy number variation, gene expression and miRNA levels in over 400 samples of HCC provided by TCGA. We employed and further developed the “cluster of cluster” method to analyze the data. We identified five subgroups according to the improved method, and the subgroups had distinct survival rates, clinical observations and molecular signatures. Subsequently, we combined somatic mutation data for these samples with the subgroups. The mutation landscapes of several important HCC-related genes were significantly different among the subgroups, including TP53, CTNNB1, BAP1, MUC4 and SAGE1. The subgroups were significantly correlated with clinical observations. Distinct molecular signatures identifying each subgroup were summarized, including telomere demethylation, specific gene promoter methylation, PRSS/PRSS2P, 8q24 amplification, UGT2B17 deletion, prognostic miRNA/mRNA expression, and different protein expression levels.

## Materials and Methods

### Copy number variation (CNV) and DNA methylation data pre-analysis

The available samples in each platform used in our study are listed in S1 Table, and the workflow pre-processing of the raw data from TCGA is shown in S1 Fig. Copy number variation data generated by microarray (level 3) were acquired from TCGA. Because the HCC tumor is a mixture of cell linages, we used the log2 transformed segment mean directly instead of transforming with thresholds. We calculated the average copy-number score of the genetic interval of genes, and we considered the score to be the copy number alteration of these genes. To avoid the bias caused by extreme CNV score values, we replaced log2 transformed scores >2 by 2 and those <-2 by -2. Then, the standard deviation (SD) of copy number variation among all samples for each gene was calculated. Genes with a SD value >0.2 were retained for further analysis.

We downloaded DNA methylation data from TCGA and combined 375 samples (level 3) to construct a matrix based on the CpG sites. Loci with NA (not available) in any of these samples were removed. We calculated the standard deviation (SD) for each locus and retained the top 10000 CpG sites with the highest SD values for further analysis.

### mRNA and miRNA pre-analysis

An expression matrix was built with 269 samples from gene scaled estimates (level 3), and only genes that were detected in all samples were retained for further analysis. We then log2 transformed the expression levels. Values less than -26 were replaced with -26 (bottom 0.1%). The standard deviation (SD) of every gene was calculated. The top 6000 genes with the highest SD values were selected for further analysis. After estimating an expression value by RPM, miRNAs (level 3) that were detected in all samples were retained for further analysis after log 2 transformation. Values less than 0 were replaced with 0 (bottom 0.1%). The top 80 miRNAs with the highest SD values were used.

### Mutation and protein level

Mutations that were called and curated (level 2) by TCGA were downloaded. The mutation matrix with samples/genes as columns/rows were constructed (1 as mutated and 0 as non-mutated). The normalized protein expression levels (level 3) evaluated by microarray were downloaded from TCGA.

### Cluster of cluster (COC) and other analysis

The COC were performed with the following steps

1. Divide mRNA into 2 to 15 sub- clusters: 2 to 15 for CNV, 2 to 7 for miRNA, and 2 to 15 for methylation sites;
2. Divide samples into 2 groups according to the features in sub- clusters on platforms [15];
3. If the one of sample size of the 2 groups is less than 10, replace the values of the features in sub- clusters with NA (not available) and return back to step 2; if not, divide the next sub- cluster;
4. Rename the values of samples on sub- clusters with 0 or 1;
5. Combine the sub- clusters to form a matrix. The row represents sub- clusters and column as samples;
6. Divide samples with matrix constructed in step 5 into 2 to 7 subgroups;
7. Calculate the survival difference of subgroups. If significant, store the sub- cluster number of platforms; if not, discard the combination;
8. Calculate the recurrence of sub- cluster number of mRNA, miRNA, CNV and DNA methylation, and select the most recurrent for further analysis;
9. Divide the samples into several groups according to “ConsensusClusterPlus”.

The analysis was performed by R (https://www.r-project.org/). The circos plot was drawn using the R package “RCircos” [16, 17], and the gene location was displayed with UCSC genome browser (http://genome.ucsc.edu/).

## Results

### Cluster of cluster (COC) analysis of HCC samples

**Fig 1:**
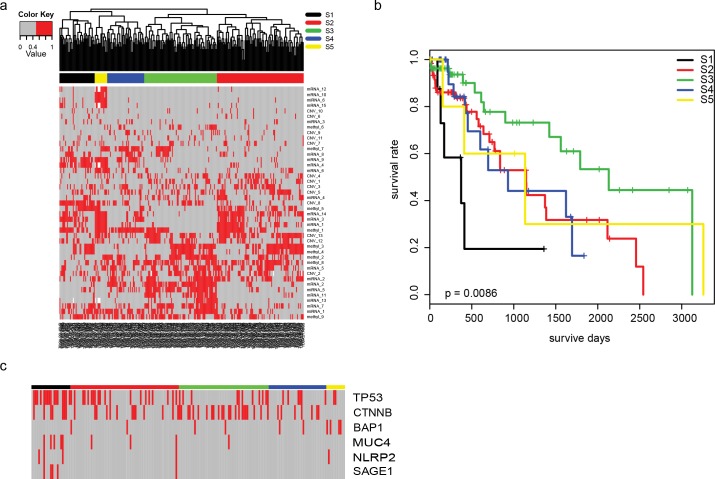
Cluster of cluster analysis of HCC samples. The a) heatmap of subclustersfronplatformed were analyzed and subgroups were identified. We found that the b) survival rate of subgroups were significantly different, and the c) mutation of some genes, especially TP53 and CTNNB1.

In previous studies, “cluster of cluster” has proven to be a powerful tool to integrate multiple “-omics” data. The contribution of each platform is determined by the number of clusters used in COC. The number of clusters of each platform depends on the stability of clusters of each platform. However, the mathematic stability is not equivalent to biological stability. We hypothesized that samples with different pathogenesis could be correlated with clinical observations, especially survival. Therefore, we developed a new way to select the most suitable number of clusters in each platform. Using this method, we found that when the number of sub- clusters of CNV/mRNA/miRNA/methylation were 13/15/6/9, respectively, the classification result was most suitable (S2A–S2D Fig, Fig 1A). The most stable number of subgroups was five (termed S1-S5) using the R package “ConsensusClusterPlus” [18] (S2E Fig). We used leave one out cross validation (LOOCV) for subsampling, and the overall accuracy reached 81.3%. The principle component analysis of the COC analysis revealed that the first four main principle components clearly distinguish the five subgroups (S3 Fig). We noted that the S1 subgroup had the worst survival rate (Fig 1B), with 20% of the subjects having a one-year survival rate, and the S3 subgroup had the best survival rate, with 90% of the subjects having a one-year survival rate and over 50% having a five-year survival rate. The survival rates of the other three subgroups (S2, S4, and S5) were comparable, with 30% of the subjects having a five-year survival rate. To further validate the classification, we looked into the somatic mutations of HCC related genes from each sample (Fig 1C). We observed that the mutational rate of TP53 was higher in S1 (16/23, 70%) than in the other subgroups (39/162, 24%, p = 3e-4). Additionally, the mutation rate of CTNNB1 in S3 (23/53, 43%) was significantly higher than in the other subgroups (27/132, 20%, p = 0.008). The mutation rate of MUC4, NLRP2, and SAGE1 in S1 was significantly higher than in the other subgroups (p = 0.008/0.008/0.003, respectively), and no mutations of BAP1 were detected in S1 (p = 0.003).

The original COC method divided these CNV/mRNA/miRNA/methylation features into 4/5/5/6 sub- clusters. When using the original COC method, each subgroup had distinct characteristics, but the clinical information and important mutations of subgroups were not significantly different from each other (data not shown), which indicated that our developed method performs better than the original COC method and suggests that the method is more powerful.

### Clinical observations and subgroups

To better interpret our classification results, we correlated the subgroups with clinical information. We found that gender, alcohol consumption, alpha-fetoprotein (AFP) level, and the AJCC (American Joint Committee on Cancer) staging level of the primary tumor were factors associated with these subgroups (Table 1). There were more female patients in S4 and S5 (60% of total) in contrast to the other subgroups (32% of the total, p = 0.0037). Patients in S1 and S5 were more likely to be involved in alcohol abuse. Fifty-seven percent of patients in S1 and S5 consumed alcohol, whereas the overall ratio was 30% in the other subgroups. Consistent with the better survival rate of S3, the sample proportion of patients with a high AFP level (>20) in this subgroup was less than in the other subgroups (24% compared to 54%, p = 0.006). Because almost all of the samples in the AJCC staging system were N0 and M0, we chose the primary tumor stage for comparison. A higher primary tumor stage for S1 and S5 was detected, whereas samples in S2 and S3 were at a relatively lower stage (p = 0.0038). These results indicate that our molecular classification results are consistent with clinical observations.

**Table 1:** Association between clinical observation and subgroups.

	S1	S2	S3	S4	S5	P val
**Gender**						**0.00365**
Female	2	27	18	19	8	
Male	14	45	39	15	3	
**Alcohol**						**0.01115**
NO	7	41	41	25	2	
YES	5	26	13	7	7	
**Child_pugh**						0.1343
Low(1)	4	31	30	15	6	
High(>1)	3	5	2	2	0	
**AFP**						**0.00572**
Low(<20)	4	27	31	9	3	
High(>20)	8	23	10	17	2	
**AJCC stage**						**0.00378**
1	2	34	30	13	1	
>1	14	37	26	21	10	
**HCV**						0.635237
No	0	4	4	3	0	
Yes	12	27	24	15	8	
**Vascular invasion**						0.234197
NO	11	44	31	19	8	
YES	2	22	20	13	1	

### Multiple alterations observed in S1

**Fig 2:**
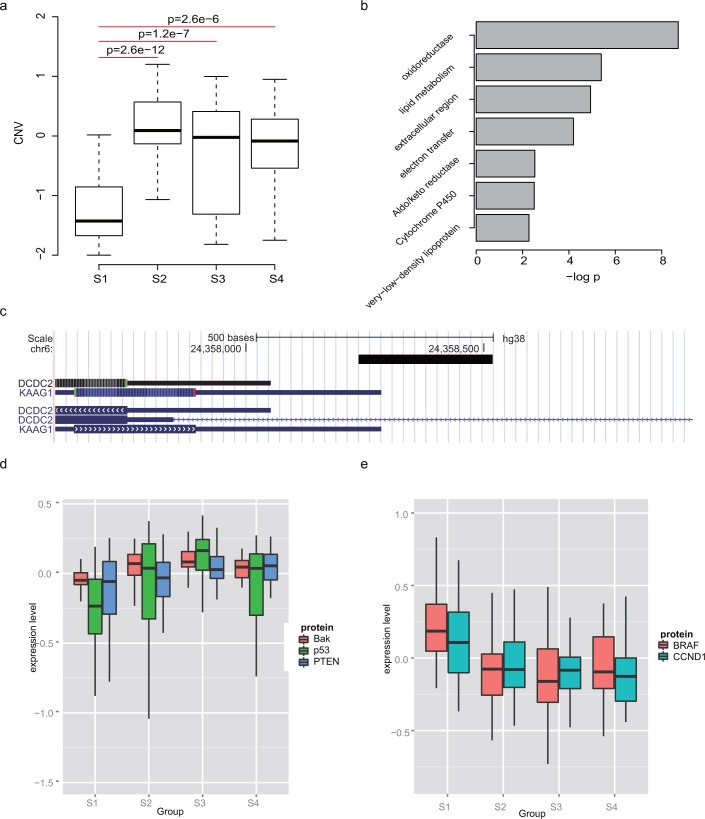
Multiple alterations were observed in S1. UGT2B17 deletion is significantly severe in S1 than S2-S4. Subcluster mRNA4 is enriched in S1, and b) mRNA4 genes were enriched in GO terms involved in lipid metabolism. On methylation level, c) DCDC gene promoter region were hypomethylated, as indicted by the black rectangle. Onco-proteins were d) highly expressed in S1, while e) tumor suppressor proteins were lowly expressed in S1.

We analyzed the molecular signatures of these subgroups. Due to the small sample size of S5, we first analyzed S1-S4. In general, we noticed that mRNA4, mRNA9, CNV8, mRNA11, mRNA13 and CNV9 were enriched in S1. Deletions of UGT2B17 (Fig 2A), PRSS2/PRSS3P2, and GSTT1/GSTTP2 (S3A–S3B Fig) were frequently observed in S1, and other reports indicate that these genes are frequently deleted in cancer cells. Many samples in this subgroup also showed amplification at 8q24. In addition, this subgroup had relatively low levels of enzymes involved in lipid metabolism and oxidation (mRNA4, Figs 1A and 2B). S1 samples also exhibited a relatively higher expression level of genes involved in extracellular matrix interaction (S2 Table, mRNA11). Hypomethylation was also detected at the promoter region of DCDC2 (doublecortin domain-containing 2, Fig 2C) in S1. According to the protein expression levels from TCGA (obtained by microarray), relative expression of tumor suppressor genes, including PTEN, TP53, and BAK, was lower in S1 compared to S2/3/4 (Fig 2D). Meanwhile, oncogenes including BRAF and CCND1 were highly expressed in S1 at the protein level (Fig 2E). The mutation rate of TP53 in this subgroup was 70% (Fig 1C). These results indicate that S1 is altered at the genetic, epigenetic, transcriptomic, and protein levels, consistent with the lower survival rate of patients in this subgroup.

### Telomere hypomethylation in S2 and S3

**Fig 3:**
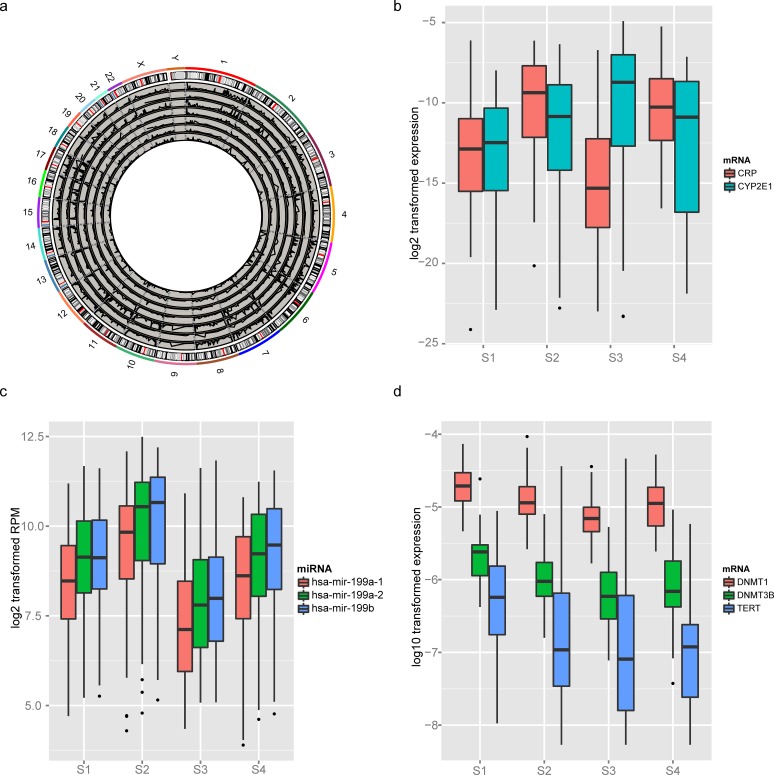
Telomere hypo-methylation was a characteristic of S2 and S3. a) The distribution of methylation clusters. Among them, Methyl1/2/6/7 were hypermethyalted, the others were hypomethylated (shown in Fig 1A). Prognostic marker b) CRP/CYP2E1, and c) miRNA mir-199a-1/mir-199a-2/mir-199b were significantly different in S2 and S3. Consistent with the hypomethylation of telomere, enzymes involved in d) telomere elongation and methylation were down regulated.

Subgroup specific copy number variations were not detected in S2 or S3. Cell cycle, extracellular related genes (mRNA3), and plasma genes (mRNA15) were altered in S2 at the transcriptome level (see GO analysis in S3 and S4 Tables). Alterations of DNA methylation in this subgroup were also observed (Methyl3 and Methyl4, Fig 1A). Hypomethylation of 6q27, 7q36.3, 10p15.3, 10q26.3 17p13.2 and 17q25.3 was characteristic of Methyl3 and Methyl4. These locations are all terminal regions of chromosomes (Fig 3A). Because DNA hypermethylation on telomere regions is an indicator of chromosomal stability, the demethylation of the telomere DNA sequences is suggestive of relatively unstable chromosomes.

Although we did not detect any specific copy number alterations in S3, specific mRNA, miRNA and DNA methylation clusters in this subgroup frequently observed. On the transcriptome level, genes involved in cell cycle and extracellular matrix adhesion (Fig 1A, S3 and S5 Tables for mRNA3 and mRNA2) were up-regulated while lipid metabolism and oxidation related genes were down-regulated (S6 Table for mRNA4). In addition, HCC diagnostic and prognostic markers CRP and CYP2E1 had decreased or increased expression, respectively, in this subgroup (Fig 3B). Additionally, prognostic microRNAs including hsa-mir-199a-1/199a-2/199b were lowly expressed in this subgroup in comparison to the other groups (Fig 3C, p<1e-7), especially S2. DNA hypomethylation of the terminal regions in S3 was even more obvious than in S2 (Fig 3A), indicating greater instability in the telomere region of chromosomes in S3. Telomerase reverse transcriptase (TERT) is a well-known gene for telomere elongation. The expression level of TERT was significantly lower in S3 in comparison to the other subgroups (Fig 3D), consistent with hypomethylation at the end of chromosomes. We also detected that the key enzymes for DNA methylation maintenance, DNMT1 and DNMT3B, had significantly decreased expression in S3 (Fig 3D). Additionally, hypermethylation on Methy5 and Methyl6 were also observed. At the genetic level, the CTNNB1 mutation rate in this group was high (43%). These result suggest that despite the high mutation rate of CTNNB1, the shortened telomeres and positive prognosis biomarkers contribute to a better survival rate in the S3 subgroup.

### Molecular characteristics of S4

**Fig 4:**
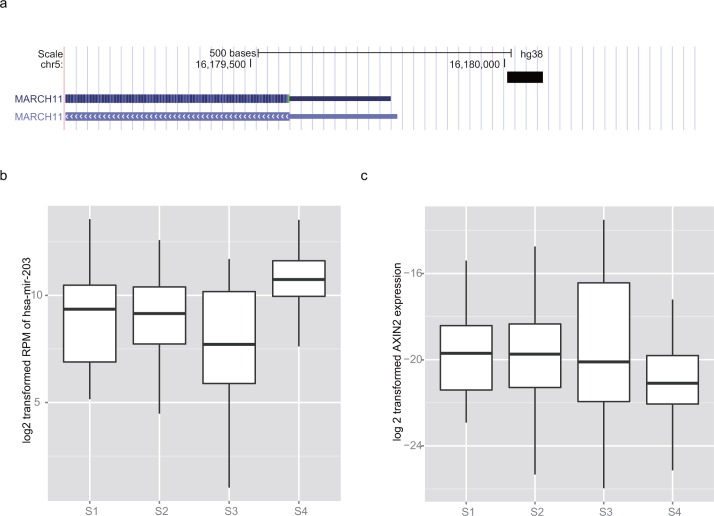
Molecular characters of S4. a) Promoter hypermethylation of MARCH11 is detected in S4. On transcriptomic level, prognostic marker b) mir-203 and c) AXIN2 were highly/lowly expressed in S4, respectively.

Copy number variations of many genes (especially amplification on 8q24 and PRSS2/PRSS3P2 deletion) were common in S4 (S4 Fig). Previous studies have indicated that amplification on 8q24 is common in HCC samples, but a significantly higher mutation rate (80%) was observed in S4 in comparison to other subgroups. Transcriptomic specific cluster features were not detected in S4. However, unlike the other groups, the epigenetic changes in S4 were sub- cluster Methyl1 and Methyl7. Hypermethylation of the promoter region of MARCH11, an E3 ligase, was observed in Methyl7 (Fig 4A). MicroRNAs including mir-203 were highly expressed in this subgroup (Fig 4B), whereas the gene AXIN2 was less expressed in S4 than in the other subgroups (Fig 4C). We also detected that the protein expression level of PTEN in S4 was significantly higher than those in the other groups at the protein level, whereas the expression of CCND1 was low (Fig 2D and 2E).

### Molecular characteristics of S5

**Fig 5:**
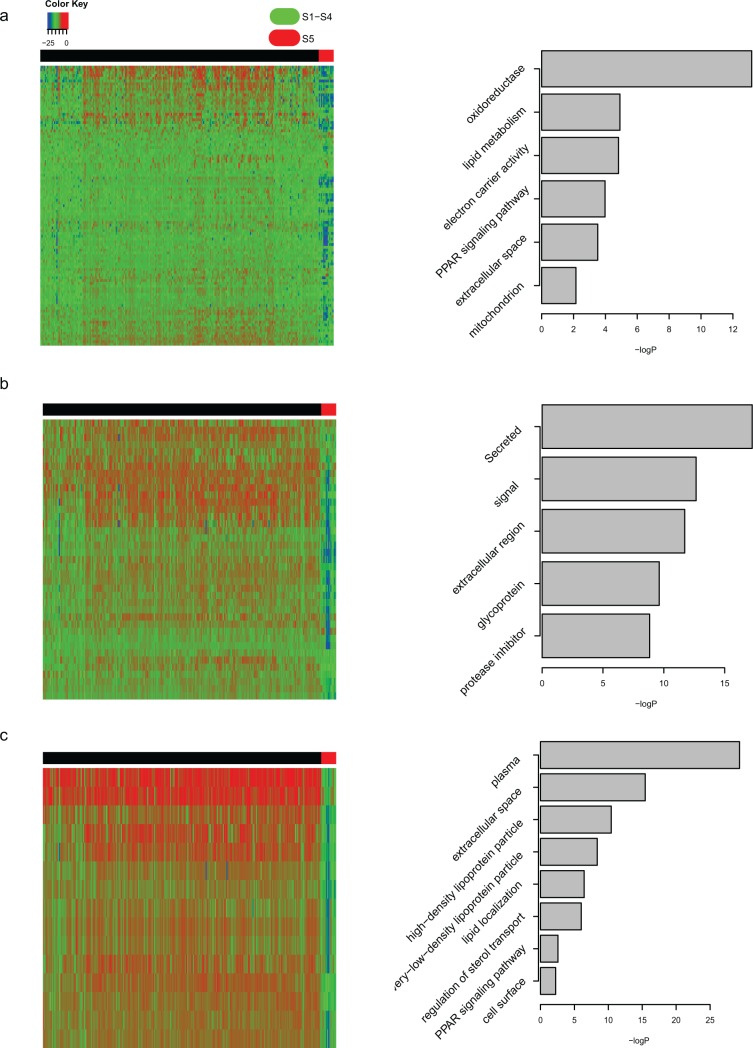
Expression level of mRNA10/12/15 and GO term of the included genes. The expression heat map of mRNA10/12/15 cluster (a-c, left panel) and the rasult of GO analysis of the related genes (a-c, right panel).

Due to the small sample size of S5, the important characteristics of S5, including the protein levels of TP53/BAK/PTEN/CCND1, were analyzed across S1 to S4 (data not shown). The most important signatures of S5 were clusters mRNA10, mRNA12, mRNA15, and miRNA6 (Fig 1A), and the related features had lower concentrations in S5 (Fig 5A–5C). The mRNA10-associated genes were enriched in multiple metabolic pathways, especially oxidoreductase (Fig 5A). The mRNA12 and mRNA15 genes were enriched in extracellular matrix terms (Fig 5B–5C), although the features of S5 occurred rarely on the other platforms. These results suggest that the alteration of S5 is limited to the transcriptomic level.

### Biomarkers of each subgroup

The multiple types of biological data comprehensively reveal the alterations characteristic of the individual subtypes (Table 2). However, this type of classification is not sufficient for application to clinical studies. To facilitate the differentiation of the subgroups, we evaluated DNA methylation, miRNA, and mRNA data to find biomarkers to distinguish these subgroups with area under curve (AUC) of a receiver operating characteristic (ROC) curve (Table 3). We found that MYBL2 (gene), SMOC2 (gene), SLC1A5 (gene), cg27046920 (CpG loci), and F2 (gene) were effective biomarkers of S1, S2, S3, S4 and S5, respectively, with relatively high AUC for each subtype, indicating the diagnostic efficacy of these genes.

**Table 2:** Summary of molecular characters of S1-S4.

	S1	S2	S3	S4	S5
**Clinical**	worst survival	medium survival	Best survival	medium survival	median survival
**CNV**	UGT2B17/PRSS2/PRSS3P2 deletion, 8q24 amplication	NA	NA	8q24 amplication	NA
**Methylation**	DCDC2 promoter hypomethylation	telemere hypomethylation	more telemere hypomethylation	MARCH11 promoter hypermethylation	NA
**mRNA**	low expression of lipid metabolism and oxidation genes	highly expressed cell cycle genes	lowly expressed cell cycle genes,CRP and highly expressed CYP2E1	Lowly expressed AXIN2	low expression of oxidoreductase, extracellular matrix, and PPAR pathway
**miRNA**	NA	highly 199a-1,199a-2,199b	lowly 199a-1,199a-2,199b	high miRNA 203	low expression of hsa-mir-194-1, hsa-mir-194-2, hsa-mir-192,hsa-mir-122
**mutation**	High TP53 mutation	NA	high CTNNB mutation	NA	NA
**protein**	High BRAF/CCND1; low TP53/CTNNB/BAK	NA	Low BRAF/CCND1; high TP53/CTNNB/BAK	low CCND1	NA

**Table 3:** The AUC of character features for each subgroup.

Gene Name	Entrez	S1	S2	S3	S4	S5
MYBL2	4605	**0.866716**	0.509491	0.779459	0.568002	0.632479
SMOC2	64094	0.609527	**0.784682**	0.756944	0.504313	0.528965
SLC1A5	6510	0.794274	0.61019	**0.882749**	0.524755	0.812599
cg27046920	/	0.591139	0.677123	0.540643	**0.859033**	0.789807
F2	2147	0.650253	0.57023	0.601754	0.541061	**0.99905**

## Discussion

The integration of multiple biological levels of data facilitates cancer classification, especially for highly heterogeneous cancer. In our work, we integrated the copy number variation, DNA methylation, gene expression and microRNA expression in more than 400 samples. Among them, all data for multiple biological levels were available for 256 samples. We performed “cluster of cluster” analysis of these samples, and five distinct subgroups were identified. We validated the findings with cross-validation and PCA analysis, and the differences between subtypes were also detected at the genetic mutation level. Each subgroup had molecular signatures that were either previously reported or newly found. Consistent with this result, the classification is associated with gender, alcohol intake, AFP level, and the AJCC staging system.

Altered features in most samples include the amplification of the 8q24 genes HLA-DQB/DQB and the overexpression of IGF2/H19. Among the samples, 8q24 is a frequently amplified locus containing a set of oncogenes in many cancer types [19, 20]. Increased expression of H19/IGF2 was observed in our study, as in previous reports [21, 22].

We noted that UGT2B17, an enzyme that catalyzes the transfer of glucuronic acid from uridine diphosphoglucuronic acid to a variety of substrates, was significantly deleted in S1. UGT2B17 is a frequently reported gene in various cancers and is frequently associated with polymorphism deletion. According to previous reports, up to 44% of the patients had a deletion of UGT2B17 in Chinese HBV affected HCC samples [4], and deletion of this gene was reported to be associated with increased prostate cancer risk, TP53 mutation, and relapse of head and neck carcinoma. Additionally, hypermethylation of the DCDC2 promoter was detected in S1. DCDC2 is a candidate tumor suppressor gene and is associated with poor prognosis [23]. In addition, well-known tumor suppressor genes including PTEN and TP53 were decreased, whereas oncogenes were highly expressed in S1. The instability of the genome, epigenome, transcriptome, miRNome and proteome makes the S1 survival rate lower than the other subgroups, which suggests that the pathology of this subtype may be a top-down dysfunction of multi “-omics”.

Both S2 and S3 had hypomethylated chromosome terminal regions and lower TERT and DNMT1/3B expression levels (as observed in Methyl3 and Methyl4). However, the hypomethylation of Methyl5/6 was also identified in S3, which suggests that S3 is less stable. Some transcriptomic characteristic genes were associated with poor prognosis, including increased CYP2E and attenuated CRP and other genes involved in the cell cycle. According to a previous study, CYP2E1 is decreased in HCC, and the overexpression of CYP2E1 [24] can induce apoptosis of HCC cell lines [25]. A high CRP expression level is associated with poor prognosis and promotes portal vein invasion in HCC [26, 27]. TERT and DNMT1/3b expression is significantly lower than in the other groups. The hypomethylation of telomeres and lack of enzymes to elongate telomeres causes cell death. Cells with low DNMT1/3B expression are prone to apoptosis, which is consistent with the result that the tumor size (AJCC T stage) was relatively smaller than in the other groups, leading to a better survival rate. Micro-RNAs, including has-mir-199a/b, which has been reported to be a poor prognosis marker in HCC, were also expressed at a low level in S3 [28]. One of the targets of hsa-mir-199a/b is FZD7, the most important WNT receptor in cancer development and progression [29]. Additionally, the mutation rate of CTNNB1 in S3 was significantly higher than that of the other subgroups. CTNNB1 mutation was correlated with a favorable prognosis of HCC according to previous reports [30,31]. We suspect that the underlying pathology of S3 may be the activation of WNT signaling pathways, thus bypassing telomerase activation and other prognostic biomarkers, which makes the overall survival rate of S3 higher. S2 may activate or suppress other important pathways, including cell cycle-related genes, and to some extent, bypasses telomerase.

The number of distinct molecular signatures in S4 was much lower than those of the other groups. However, we still noticed that in S4, miRNA 203, a tumor suppressor gene that is involved in invasion and migration [32] and is down-regulated and activates many targets during the carcinogenesis of HCC[33,34], was highly expressed in S4. AXIN2, a key gene in the WNT signaling pathway, had low expression in S4.

Taken together, our integration of multiple biological data levels revealed the landscape of heterogeneity of HCC and successfully classified subtypes that are associated with cancer survival by their molecular signatures.

## Supporting Information

S1 Fig Work flow of this work. (TIFF)

S2 Fig Density of a-d) CNV/mRNA/miRNA/methylation in COC analysis and e) “ConsensusClusterPlus” analysis show that the subgroup is stable when the number reached 5.
(TIFF)

S3 Fig PCA analysis of subgroups. The subgroups can be divided by the first four main components.
(TIF)

S4 Fig Copy numbers of a) PRSS2/PRSS3P2 and b) GSTT1/GSTTP2 in S1-S4.
(EPS)

S1 Table Samples used for analysis in each platform. And for integrated study, samples presented in all these four platforms were used.
(XLSX)

S2 Table GO analysis result of mRNA11 genes. (XLSX)

S3 Table GO analysis result of mRNA3 genes. (XLSX)

S4 Table GO analysis result of mRNA15 genes. (XLSX)

S5 Table GO analysis result of mRNA2 genes. (XLSX)

S6 Table GO analysis result of mRNA4 genes. (XLSX)
