# Supplementary figures and images for: Integrated Multiple “-omics” Data Reveal Subtypes of Hepatocellular Carcinoma
Source: PLoS One. 2016 Nov 2;11(11):e0165457. doi: 10.1371/journal.pone.0165457 (PMC5091875; doi:10.1371/journal.pone.0165457)

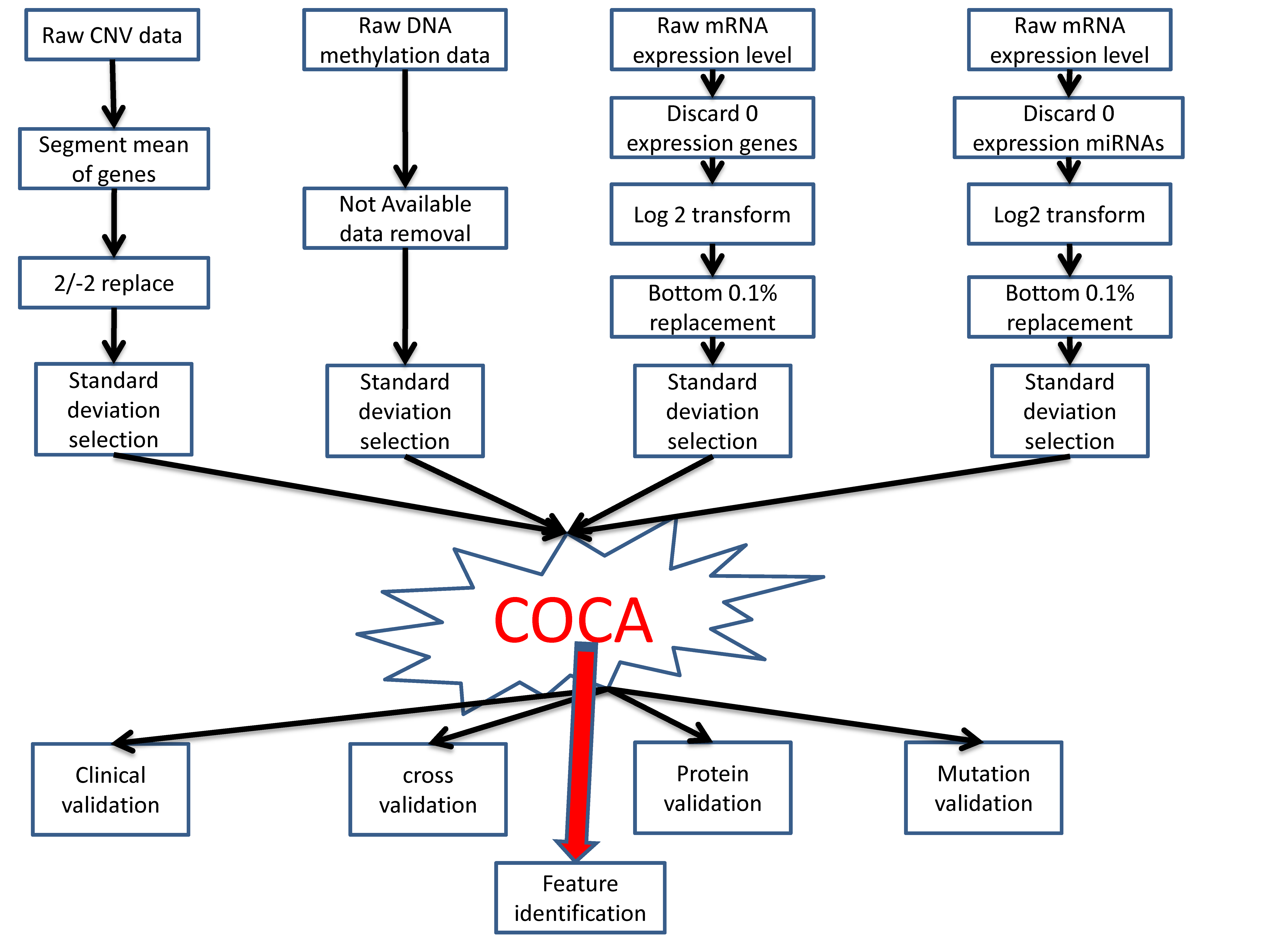

Supplement: S1 Fig — (TIFF) [file pone.0165457.s001.tiff]

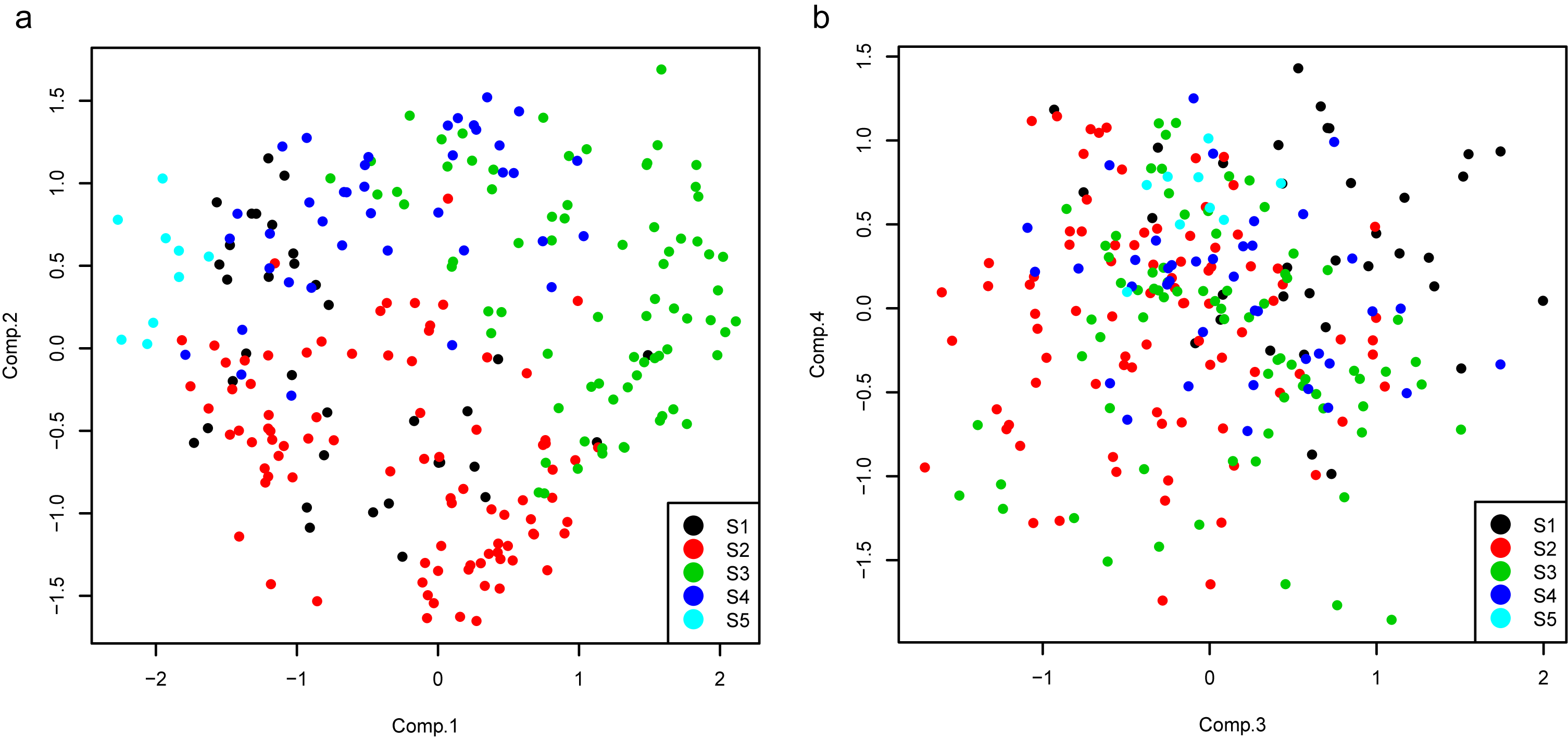

Supplement: S3 Fig — The subgroups can be divided by the first four main components. (TIF) [file pone.0165457.s003.tif]
